# Metabolism Supports Macrophage Activation

**DOI:** 10.3389/fimmu.2017.00061

**Published:** 2017-01-31

**Authors:** P. Kent Langston, Munehiko Shibata, Tiffany Horng

**Affiliations:** ^1^Department of Genetics and Complex Diseases, Harvard T.H. Chan School of Public Health, Boston, MA, USA

**Keywords:** macrophage, macrophage metabolism, macrophage activation, immunometabolism, mitochondria, electron transport chain, mTOR, AKT

## Abstract

Macrophages are found in most tissues of the body, where they have tissue- and context-dependent roles in maintaining homeostasis as well as coordinating adaptive responses to various stresses. Their capacity for specialized functions is controlled by polarizing signals, which activate macrophages by upregulating transcriptional programs that encode distinct effector functions. An important conceptual advance in the field of macrophage biology, emerging from recent studies, is that macrophage activation is critically supported by metabolic shifts. Metabolic shifts fuel multiple aspects of macrophage activation, and preventing these shifts impairs appropriate activation. These findings raise the exciting possibility that macrophage functions in various contexts could be regulated by manipulating their metabolism. Here, we review the rapidly evolving field of macrophage metabolism, discussing how polarizing signals trigger metabolic shifts and how these shifts enable appropriate activation and sustain effector activities. We also discuss recent studies indicating that the mitochondria are central hubs in inflammatory macrophage activation.

## Overview of Macrophage Activation

Macrophages are activated by various signals to acquire specialized functions (1, 2). During microbial infection, macrophages are activated by pathogen-associated molecular patterns such as LPS to an inflammatory phenotype that is characterized by production of inflammatory cytokines and induction of antimicrobial activities. In the context of type II immune responses, they are activated by the cytokines IL-4 and IL-13 to upregulate tissue repair and immunoregulatory activities. LPS- or IL-4-activated macrophages [hereafter referred to as M(LPS) and M(IL-4) macrophages, respectively] (3) have served as a paradigm for studies of macrophage activation and metabolism and will be discussed in detail in this review (1, 2).

Polarizing signals upregulate transcriptional programs to enforce macrophage activation (1, 2). In M(LPS) macrophages, LPS engages TLR4 to trigger a signaling pathway that culminates in the activation of transcription factors such as NF-κB and IRFs. This allows for the induction of a transcriptional program that encodes hallmark genes such as *Il1b, Il6*, and *TNFa*. In M(IL-4) macrophages, IL-4 binds the IL-4 receptor to trigger a JAK–STAT pathway that activates STAT6, the transcription factor responsible for induction of signature genes such as *Retnla, Arg1*, and *Chil3*. Macrophages lacking NF-κB or STAT6 (or upstream signaling components) are profoundly impaired in M(LPS) and M(IL-4) activation, respectively, underscoring the transcriptional basis of macrophage activation (1, 2).

## Regulation of Metabolic Shifts by Polarizing Signals

While metabolic shifts are triggered by growth factor signaling and nutrient availability in all mammalian cells, polarizing signals have a critical and prominent role during macrophage activation. In addition to the canonical signaling pathways discussed above (e.g., NF-κB, JAK–STAT), polarizing signals impinge on metabolic signaling pathways, enabling coordinate induction of effector activities and the metabolic processes needed to sustain those activities.

**Figure 1:**
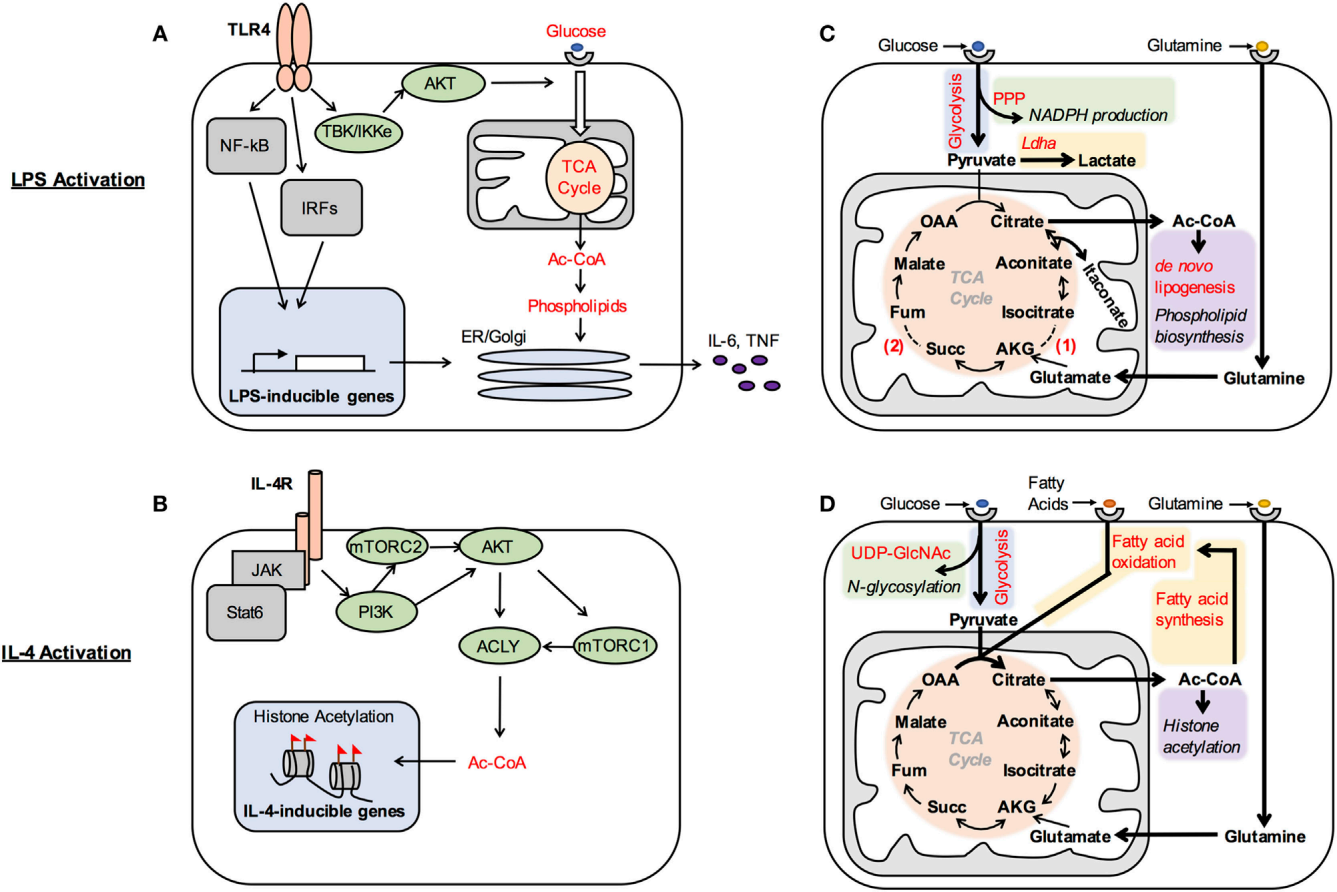
**Metabolic control of macrophage activation**. **(A,B)** Metabolic signaling pathways are activated by polarizing signals to coordinate metabolic support of macrophage activation. Akt is activated by TBK/IKKe in LPS-stimulated dendritic cells **(A)**, while IL-4R signaling impinges on PI3K to activate mTORC2, Akt, and mTORC1 in M(IL-4) macrophages **(B)**. One consequence of LPS-mediated Akt activation is increased glucose oxidation. This supports production of phospholipids, which allows for expansion of the secretory compartment for elaboration of high levels of proinflammatory cytokines **(A)**. In M(IL-4) macrophages, one consequence of Akt–mTORC1 activation is to increase Acly expression and activity. This enhances production of a cytosolic/nuclear pool of Ac-CoA, which regulates histone acetylation at a subset of IL-4-inducible genes **(B)**. Note that this figure illustrates what is currently known regarding the major metabolic targets of Akt and mTOR in their control of M(LPS) and M(IL-4) activation, and that additional targets will undoubtedly emerge in future studies. **(C,D)** Metabolic reprograming regulates macrophages activation and function. **(C)** M(LPS) activation is associated with increased aerobic glycolysis to generate high levels of lactate and associated with pentose phosphate pathway (PPP) activity to make NADPH and nucleotides. Citrate is used for Ac-CoA and phospholipid production to support increased cytokine secretion and also gives rise to the antimicrobial species itaconate. Downregulation of isocitrate dehydrogenase expression (1) and inhibition of succinate dehydrogenase by itaconate (2) disrupt the TCA cycle in M(LPS) macrophages, requiring glutaminolysis and the arginosuccinate shunt (not shown) to provide α-ketoglutarate (AKG) and fumarate (fum) to replenish the cycle. **(D)** M(IL-4) activation is associated with increased utilization of glucose, fatty acids, and glutamine. Glucose goes to support N-glycosylation through UDP-GlcNAc. Multiple carbon substrates, including glucose, fatty acids, and glutamine, drive increased TCA cycle activity to boost histone acetylation. β-Oxidation is fueled by fatty acids that are either imported or synthesized *de novo* from glucose. Succ, succinate; OAA, oxaloacetate.

Metabolic signaling pathways activated by polarizing signals include Akt, mTORC1, mTORC2, and AMPK (Box 1). In M(IL-4) macrophages, Akt and mTORC1 form a coordinated signaling module that is activated by PI3K and mTORC2 (4–6). Downstream of LPS/TLR4, Akt can be activated by TBK/IKKe in addition to PI3K, and Akt activity can be disassociated from mTORC1 activity (7) (Figures 1A,B). Therefore, Akt and mTORC1 activities are regulated disparately in M(LPS) and M(IL-4) macrophages with the potential for differential control of metabolism and function. IL-10, which activates macrophages to an anti-inflammatory phenotype, stimulates AMPK activity, while LPS signaling diminishes AMPK activation (8).

Box 1 **Glossary of major metabolic regulators and pathways discussed in text.**

*mTOR*: mTOR kinase is found in two complexes in mammals, mTORC1 and mTORC2. Other subunits of these complexes are unique to and define the complexes, such as Raptor and Rictor in mTORC1 and mTORC2, respectively. mTORC1 links availability of nutrients (in particular amino acids) and growth factor signaling to anabolic processes such as macromolecule synthesis and nutrient storage in proliferating cells and tumor cells. mTORC2 phosphorylates and activates Akt and other kinases of the AGC superfamily to control metabolism, survival, and cytoskeletal organization.

*Akt*: Akt kinases regulate cell survival, metabolism, and cytoskeleton. Downstream of growth factor receptors and IL-4R, Akt is activated by PI3K signaling. This is due, at least in part, to PI3K-mediated activation of mTORC2, which critically phosphorylates Akt on S473. Activated Akt phosphorylates the TSC complex, a negative regulator of mTORC1, to stimulate mTORC1 activity. Therefore, the activities of Akt, mTORC1, and mTORC2 are intricately linked in the IL-4 signaling pathway.

*AMPK*: AMPK is a key regulator of cellular energy homeostasis. In response to an increasing ADP to ATP ratio, AMPK stimulates ATP generating processes (e.g., fatty acid oxidation) while inhibiting non-critical ATP consuming processes (e.g., lipid synthesis) to restore energy balance.

*Glycolysis*: glycolysis is the process by which glucose is incompletely oxidized in the cytosol, yielding lactate as its final product. Compared to oxidative metabolism, glycolysis is fast but energy inefficient.

*Pentose phosphate pathway*: this is a shunt of glycolysis that produces NADPH, important for maintaining cellular redox balance, and nucleotides.

*Hexosamine pathway*: this is a glycolytic shunt that produces UDP-GlcNAC, the metabolic substrate for N-glycosylation and O-GlcNAcylation modifications of proteins.

*TCA cycle*: carbon substrates such as glucose-derived pyruvate, fatty acids, and glutamine can be oxidized in the TCA cycle. This mitochondrial process generates the reducing equivalents NADH and FADH2, which fuels the electron transport chain (ETC) to generate ATP *via* oxidative phosphorylation. TCA cycle intermediates can also be diverted for other purposes (e.g., cytosolic production of Ac-CoA) or serve other cellular functions (e.g., regulating the activities of chromatin modifying enzymes).

*Ac-CoA*: Ac-CoA is a two-carbon metabolite that partitions into two major pools in the cell, mitochondrial versus nuclear/cytoplasmic. The latter pool contributes to histone acetylation and lipid synthesis and is critically regulated by the enzyme Acly, which cleaves mitochondria-derived citrate to produce Ac-CoA.

*Electron transport chain (ETC)*: the ETC consists of five complexes and two mobile electron carriers embedded in the mitochondrial membrane that link oxidation of carbon substrates to ATP production. The ETC couples the energy of electron transfer (from NADH and FADH2) to the generation of a proton motive force across the inner membrane, which is harnessed by complex V to drive ATP synthesis.

What are the consequences of regulation of these metabolic signaling pathways by polarizing signals? Akt activity drives glucose utilization, including aerobic glycolysis as well as glucose oxidation, in LPS-activated DCs and macrophages (7, 9) and M(IL-4) macrophages (10). In LPS-activated DCs, glucose oxidation supports production of cytosolic Ac-CoA to boost phospholipid synthesis, ER and Golgi expansion and secretory capacity, and proinflammatory cytokine production (Figure 1A) (7). In M(IL-4) macrophages, Akt–mTORC1 activity controls the expression and activity of Acly, which cleaves cytosolic citrate to produce a nuclear/cytoplasmic pool of Ac-CoA. Such Ac-CoA production fuels histone acetylation at a subset of IL-4-inducible genes for optimal M(IL-4) activation (Figure 1B) (10). Because Ac-CoA production occurs in the context of LPS-inducible inflammatory cytokine production and IL-4-inducible gene expression, Akt differentially supports the effector activities of LPS- and IL-4-activated dendritic cells/macrophages. In LPS-activated DCs, mTORC1 activity inhibits oxidative metabolism, apparently by stimulating production of NO (11), which can damage the ETC. Importantly, the relevant metabolic targets of Akt, mTORC1, and other metabolic signaling pathways in control of macrophage activation remain incompletely characterized and represent an important avenue of future investigation.

Consistent with important roles for metabolic signaling pathways in regulating activation, pharmacological or genetic manipulations of their activity alter macrophage activation (6). Blocking Akt, mTORC1, and mTORC2 reduces M(IL-4) activation, indicating that the activities of the Akt–mTORC1 axis and its upstream activator mTORC2 potentiate M(IL-4) activation (5, 10, 12). By contrast, AMPK activity limits the inflammatory phenotype of M(LPS) macrophages (8). This has led to the proposal that anabolic metabolism, which is regulated by Akt and mTORC1 and involves macromolecule synthesis, supports the role of macrophages in type I and type II inflammatory responses. By contrast, catabolic metabolism, which is coordinated by AMPK and involves breakdown of macromolecules, may sustain the anti-inflammatory functions of macrophages (13).

Metabolic signaling pathways integrate metabolic cues to control macrophage activation. The Akt–mTORC1 pathway couples amino acid sensing to M(IL-4) activation such that amino acid availability increases, while amino acid deficiency reduces IL-4-inducible gene induction (10, 12). Like block of the Akt–mTORC1 pathway, loss of Lamtor1, which mediates lysosomal amino acid sensing by mTORC1, impairs M(IL-4) activation (12). Therefore, physiological activation of metabolic signaling pathways supports M(IL-4) activation. However, aberrantly elevated mTORC1 activity (due to genetic deletion of its negative regulator TSC1) attenuates M(IL-4) activation by shutting down Akt signaling. In this setting, exaggeration of mTORC1-mediated feedback inhibition ultimately diminishes Akt activity to impair M(IL-4) activation (4). These findings suggest that pathophysiological elevation of mTORC1 activity corrupts the ability of metabolic signals to appropriately tune macrophage activation.

Polarizing signals can also impinge on transcriptional master regulators of metabolism to trigger metabolic shifts. For example, the nuclear receptors PPARγ and PPARδ and the transcriptional coactivator PGC1β regulate oxidative metabolism and mitochondrial biogenesis in multiple contexts. In M(IL-4) macrophages, their induction by STAT6 allows for upregulation of oxidative metabolism that is apparently needed for optimal activation (14, 15). The transcription factor IRF4 has also emerged as a target of mTORC2 and STAT6 signaling that regulates M(Il-4) activation. IRF4 is needed for induction of glycolysis in M(IL-4) macrophages, and upregulation of many glycolytic genes is reduced in IRF4-deficient BMDMs (5). In M(LPS) macrophages, increases in the protein levels and activity of the transcription factor HIF1α underlie upregulation of glycolysis (16). HIF1α is normally an unstable protein but becomes stabilized by LPS-triggered alterations to ETC activity that increases mitochondrial ROS (mtROS) production (17). Moreover, LPS signaling increases HIF1α activity by upregulating the PKM2 isoform of pyruvate kinase (18). In addition to its role in glycolysis, PKM2 is a HIF1α coactivator, which binds to HIF1α to enhance its transcriptional activity.

Therefore, polarizing signals co-opt the metabolic machinery to coordinate metabolic shifts, which are described in more detail below.

## Metabolic Shifts during Macrophage Activation

Early studies suggested that M(LPS) macrophages preferentially use glucose, while M(IL-4) macrophages consume fatty acids. M(LPS) macrophages upregulate expression of the glucose transporter Glut1 (19), while M(IL-4) macrophages increase expression of lipoprotein lipase and CD36, which mediate uptake and transport of fatty acids (14). More recent studies indicate complexity in fuel utilization, reporting enhanced consumption of glucose in M(IL-4) macrophages (5, 10) and of glutamine in M(LPS + IFNg) and M(IL-4) macrophages (20, 21). Below we discuss how M(LPS) and M(IL-4) macrophages augment carbon substrate utilization to meet increasing bioenergetic and biosynthetic demand and how they direct carbon substrates to different metabolic fates to support distinct effector activities.

### Glucose Utilization in M(LPS) Macrophages

Early studies indicated that avid glucose consumption in LPS-activated dendritic cells and macrophages sustains high rates of aerobic glycolysis, or “Warburg metabolism” (Figure 1C). Experimental attenuation of glycolysis reduces inflammatory cytokine production and bacterial killing (9, 16), indicating that glycolysis is needed for optimal elaboration of the inflammatory phenotype. Mechanistically, how M(LPS) macrophages increase glucose utilization and direct glucose carbons into aerobic glycolysis are relatively well understood. LPS signaling activates Akt to enhance glucose uptake (7), while induction of HIF1α, a master regulator of glycolysis, allows for upregulation of multiple genes to stimulate glycolysis. For example, upregulation of PFKFB3, a tissue-specific isoform of PFKFB, drives overall flux through glycolysis, while induction of PDK1, a negative regulator of pyruvate oxidation in the mitochondria, and LDHA, the enzyme that converts pyruvate into lactate, increases aerobic glycolysis (22, 23). Glycolytic production of ATP may be important to fuel optimal induction of effector activities and maintain cellular viability, given that oxidative metabolism is impaired in M(LPS) macrophages. Glucose oxidation is also important in LPS-stimulated dendritic cells and macrophages, driving Ac-coA and phospholipid synthesis and inflammatory cytokine production as discussed above (Figures 1A,C) (7). How glucose oxidation and glycolysis are coordinated is incompletely understood but seems to be a pivotal node in control of M(LPS) and M(LPS + IFN-g) activation, as various manipulations that alter this balance perturb the activation phenotype (9, 24).

Remarkably, tracing experiments indicate two “breaks” in the TCA cycle of M(LPS) and M(LPS + IFN-g) macrophages that alter glucose oxidation with profound consequences for the activation phenotype (Figure 1C) (20). The first break regulates the fate of the TCA cycle intermediate citrate, which is normally converted to α-ketoglutarate by IDH1. In these macrophages, downregulation of IDH1, but upregulation of immunoresponsive gene 1 (IRG1), an enzyme with decarboxylase activity for citrate-derived aconitase, drives the production of itaconate. By inhibiting the glyoxylate cycle, a variation of the TCA cycle found in microorganisms, itaconate has direct microbicidal activity against several pathogens (25). In addition, itaconate competitively inhibits succinate dehydrogenase (SDH) in the TCA cycle leading to the second “break” (26) (Figure 1C). Because SDH is a subunit of complex II (CII) of the ETC, itaconate production perturbs ETC activity and oxidative metabolism, and has been linked to regulation of mtROS production and inflammatory gene induction (see below). Such inhibition of SDH also leads to accumulation of its substrate succinate, of note because extracellular succinate can signal through GPR91/SUCNR1 to regulate dendritic cell migration to draining lymph nodes and antigen presentation capacity (27). Therefore, altered carbon flow through the TCA cycle critically impacts M(LPS) activation.

Arginine, which is a substrate for iNOS in NO production, will be discussed briefly here because of its link to the TCA cycle. Jha et al. report that in M(LPS + IFN-g) macrophages, part of the TCA cycle is co-opted to form an aspartate–arginosuccinate shunt in which aspartate and citrulline are used by argininosuccinate synthase (Ass1) and argininosuccinate lyase (Asl) to generate arginine (20). In the context of *Mycobacterium* infection, macrophages initially import extracellular arginine and export citrulline (a by-product of arginine metabolism by iNOS) but switch to citrulline import for arginine regeneration in this shunt only after extracellular arginine becomes depleted. Such import of extracellular arginine may allow the macrophages to limit T cell responses (28, 29).

Other key nodes in glucose utilization have been shown to control M(LPS) activation. M(LPS) macrophages increase flux through the pentose phosphate pathway (PPP), a glycolytic shunt, by downregulating the sedoheptulose kinase carbohydrate kinase-like protein CARKL. This allows for increased production of NADPH in the PPP and was proposed to provide redox balance in M(LPS) macrophages (Figure 1C). Consistently, CARKL knockdown or overexpression perturbed induction of LPS-inducible genes (30). NADPH production is also likely to be important for fueling the activities of NADPH oxidase and iNOS, which produce ROS and NO, respectively, and are important components of antimicrobial defense.

### Glutamine Utilization in M(LPS) and M(IL-4) Macrophages

An important fate of glutamine in M(LPS) and M(IL-4) macrophages appears to be anaplerosis, or refilling of the TCA cycle (20, 21). Glutamine-derived carbons enter the TCA cycle as α-ketoglutarate, replenishing carbons lost by diversion of citrate (Figures 1C,D). In M(IL-4) macrophages, this process has been linked to the induction of some IL-4-inducible genes, although the underlying mechanisms remain unclear (20). In addition, glutamine supplies nitrogen for the hexosamine pathway, a glycolytic shunt that produces UDP-GlcNAC. UDP-GlcNAC is the substrate for N-glycosylation, and block of this pathway impairs cell surface expression of some proteins expressed abundantly in M(IL-4) macrophages (Figure 1D) (20).

### Glucose Utilization in M(IL-4) Macrophages

In M(IL-4) macrophages, enhanced glucose consumption sustains glycolysis as well as glucose oxidation, although the balance of these processes is shifted toward oxidation relative to M(LPS) macrophages (Figure 1D). Glucose use is stimulated by Akt activity and IRF4 (5, 10), and fuels Ac-CoA production for histone acetylation at IL-4-inducible genes (10) and lipid synthesis (31). Glucose utilization also supports UDP-GlcNAC synthesis in the hexosamine shunt (Figure 1D) (20).

### Fatty Acid Utilization in M(IL-4) Macrophages

Early studies implicated fatty acid uptake and oxidation in IL-4-inducible gene induction in M(IL-4) macrophages (14). More recently, CD36 was shown to play a key role in delivering lipoproteins to lysosomes, where breakdown of the cargo by lysosomal lipase (LAL) liberates fatty acids that ultimately become available for β-oxidation in the mitochondria. This process was linked to the induction of several IL-4-inducible genes and markers and elimination of helminth infection. Intriguingly, fatty acid synthesis was also implicated, since blocking fatty acid synthesis reduced IL-4-inducible gene induction similar to inhibition of LAL or β-oxidation (Figure 1D) (32).

Another recent study identified a protein called FAMIN that drives *de novo* lipogenesis concurrent with fatty acid oxidation (31) in M(IL-4) macrophages. Glucose tracing experiments indicate that FAMIN promotes flux of glucose-derived carbons into lipogenesis, and that newly synthesized fatty acids are esterified to CoA and modified by acyl carnitines suggesting their utilization for β-oxidation (Figure 1D). How FAMIN regulates such carbon flux remains unknown, but FAMIN interacts with fatty acid synthase at peroxisomes. Importantly, FAMIN-deficient macrophages have aberrant NLRP3 inflammasome activation as well as reduced bacterial killing, which could be a result of attenuated mitochondrial and NADPH ROS production and/or ATP levels (31). Future studies to further characterize how fatty acid synthesis and oxidation cycles are regulated and the consequences for macrophage activation are warranted.

### Cholesterol Metabolism and Type I IFN Responses

A recent study indicated an unexpected link between cholesterol metabolism and type I IFN signaling (33). In response to type I IFN signaling, macrophages increase cholesterol import but reduce its biosynthesis. This shift supports IFN-inducible gene expression and resistance to viral infection and is coordinated by STING, a key regulator of type I IFN signaling. Because it resides at the ER where cholesterol is synthesized, STING may link sensing of cholesterol biosynthesis to type I IFN responses, thus defining a metabolic-inflammatory circuitry that regulates antiviral defense (33).

## The Mitochondria at the Center of M(LPS) Activation

**Figure 2:**
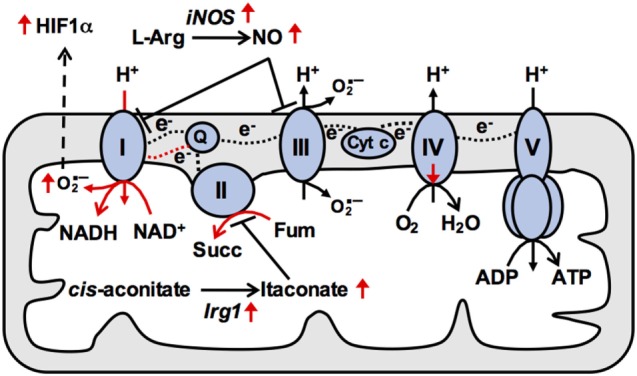
**Reduced mitochondrial respiration in M(LPS) macrophages is due to changes in activities of electron transport chain (ETC) complexes**. In naïve macrophages, electrons (e^−^) from reducing equivalents such as NADH are transferred between ETC complexes *via* mobile electron carriers and onto oxygen, generating a proton (H^+^) concentration gradient across the inner mitochondrial membrane that powers ATP production at complex V. In particular, the mobile electron carrier quinone (Q) transfers electrons from complex I (CI) and complex II (CII) to complex III, and is oxidized at complex III allowing it to return to CI and CII to repeat the cycle. In M(LPS) macrophages, increased production of nitric oxide (NO) contributes to impaired respiration. In addition, increased CII activity triggering buildup of reduced Q at complex III may provide the thermodynamic driving force allowing Q to deliver electrons to CI [reverse electron transport (RET)]. Such RET is associated with elevated superoxide production $\left({\text{O}}_{2}^{-}\right)$ at CI, leading to stabilization of HIF1α and enhanced *Il1b* expression. Therefore, mitochondrial ETC adaptations underpin M(LPS) macrophage effector functions. Cyt *c*, cytochrome *c*; succ, succinate; Fum, fumarate. Black and red dashed lines indicate forward and reverse electron transport respectively.

It has long been appreciated that stimulation of macrophages and dendritic cells with LPS or LPS + IFNg shuts down oxidative metabolism. An underpinning mechanism could be interactions of NO with iron–sulfur clusters in the ETC, since iNOS inhibition rescues oxidative metabolism (Figure 2) (34, 35). In addition, M(LPS) macrophages produce ROS from complex I (CI), which has been linked to enhanced bactericidal activity (36). The underlying mechanism involved mitochondria-localized interactions between TRAF6, a component of the TLR4 signal transduction cascade, and ECSIT, a regulator of CI assembly. More recently, a flurry of papers has further underscored the connection between oxidative metabolism, ETC activity, and inflammatory gene induction, thus positioning the mitochondria at the center of M(LPS) activation.

The IRG1/itaconate axis appears to play a key role in regulating oxidative metabolism and inflammatory gene induction in M(LPS) and M(LPS + IFN-g) macrophages (Figure 2). As discussed above, induction of IRG1 allows for itaconate production and inhibition of SDH (26). Addition of exogenous itaconate inhibits oxidative metabolism, while IRG1 deficiency enhances oxidative metabolism (37). Importantly, itaconate addition inhibits production of IL-6 and IL-12, while IRG1 deficiency augments their production (37), suggesting that the IRG1/itaconate axis serves as a built-in negative feedback that impinges on the ETC to limit excessive inflammatory responses. Another recent study also described ETC alterations during *E. coli* infection, specifically a transient increase in CII activity but a decrease in CI activity (38). Increased CII activity was triggered by the activities of NADPH oxidase and the Src kinase Fgr, which phosphorylates the SDHA subunit of CII. Like the other studies, ETC remodeling was linked to inflammatory gene induction (38).

How exactly these ETC alterations regulate inflammatory gene induction remains unclear. It has been suggested that CII activity drives reverse electron transport (RET) to potentiate mtROS production and HIF1α stabilization (17) (Figure 2). Increased CII activity fuels electron buildup at CIII, providing the thermodynamic driving force favoring backward electron flow at CI. Such RET is associated with mtROS production, which may stabilize HIF1α (39) to allow for transcriptional induction of its target *Il1b* (Figure 2) (21). In support of this model, mtROS scavengers and pharmacological inhibitors of CII reduce inflammatory gene induction (17, 37). Ectopic expression of the mitochondrial oxidase AOX, which should alleviate RET by oxidizing excess electrons, also recapitulates the phenotype (17). However, ETC activities similarly potentiate induction of inflammatory genes not thought to be regulated by HIF1α (e.g., *Il6*), so clarification of additional underpinning mechanisms remains an outstanding question.

## Integration of Metabolic Signals by Chromatin

Recent studies indicate that metabolism impinges on chromatin for transcriptional control of macrophage activation. Cheng et al. showed that priming with the *Candida albicans* product β-glucan enhances the ability of a subsequent LPS challenge to induce genes encoding inflammatory cytokines. This process is dependent on Akt and mTORC1 as well as glycolytic activity and correlates with chromatin changes at the promoters of these genes (40). As discussed above, Covarrubias et al. showed that Akt–mTORC1 signaling links amino acid sensing to control of Ac-CoA production, histone acetylation, and IL-4-inducible gene induction in M(IL-4) macrophages (10). Only a subset of IL-4-inducible genes is regulated in this manner, including genes controlling cellular proliferation and chemokine production. These findings suggest that Akt–mTORC1 signaling couples metabolic cues to chromatin to calibrate energetically demanding aspects of M(IL-4) activation (10).

## Final Thoughts and Future Directions

Here, we have focused on how metabolism supports M(LPS) and M(IL-4) activation, but macrophages can be activated to intermediate states within the M(LPS)-M(IL-4) continuum, or to other states by distinct polarizing signals. The focus on M(LPS) and M(IL-4) activation is meant to draw a contrast between two very different activation phenotypes and their metabolic underpinnings, for example, with regard to shut down versus increased oxidative metabolism in M(LPS) and M(IL-4) macrophages, respectively. In addition to further exploiting the M(LPS)/M(IL-4) paradigm to elucidate metabolic underpinnings of macrophage activation, future studies should examine additional activation states as well as tissue-specific macrophages and macrophages from different disease conditions; however, studying metabolism *in vivo* remains a major challenge.

In conclusion, we have highlighted various ways in which metabolism underpins macrophage activation. Polarizing signals impinge on metabolic signaling pathways, which coordinate biosynthetic and bioenergetic support of macrophage activation. Metabolic sensing can critically calibrate macrophage activation, as illustrated by the ability of the Akt–mTORC1 pathway to couple physiological and pathophysiological increases in nutrient availability to differential regulation of M(IL-4) activation, or STING to link cholesterol biosynthesis to type I IFN signaling. Future studies are expected to highlight the role of mitochondria in integrating metabolic cues to TCA cycle and ETC activities for control of inflammatory macrophage activation. Future studies should also uncover a prominent role for chromatin at the interface of metabolism and transcriptional control of macrophage activation.

## Author Contributions

TH, PL, and MS wrote the manuscript, and TH and PL prepared the figures.

## Conflict of Interest Statement

The authors declare that the research was conducted in the absence of any commercial or financial relationships that could be construed as a potential conflict of interest.
